# Clinical, epidemiological, and drug resistance insights into HIV-positive patients in Meizhou, China

**DOI:** 10.3389/fcimb.2023.1330826

**Published:** 2024-01-19

**Authors:** Xianhui Liu, Sandip Patil, Xuemin Guo, Feiqiu Wen, Xianyan Zhang, Zhixiong Zhong, Xinlu Wang

**Affiliations:** ^1^ Department of Laboratory Medicine, Meizhou People’s Hospital, Meizhou, Guangdong, China; ^2^ Guangdong Provincial Key Laboratory of Precision Medicine and Clinical Translation Research of Hakka Population, Meizhou, Guangdong, China; ^3^ Division of Hematology and Oncology, Shenzhen Children’s Hospital, Shenzhen, China; ^4^ Pediatric Research Institute, Shenzhen Children’s Hospital, Shenzhen, China; ^5^ Chinese Academy of Sciences (CAS) Key Laboratory of Infection and Immunity, Institute of Biophysics, Chinese Academy of Sciences, Beijing, China; ^6^ University of Chinese Academy of Sciences, Beijing, China

**Keywords:** HIV/aids, epidemiology, western blotting, drug resistance genotype, Meizhou

## Abstract

The acquired immunodeficiency syndrome (AIDS) epidemic, resulting from human immunodeficiency virus (HIV) infection, exhibits distinct regional characteristics. This study undertakes a retrospective analysis of the epidemiological and clinical features of 195 HIV-positive cases in Meizhou, China, from May 1, 2018 to December 31, 2019. Western blotting (WB) confirmed and assessed these cases. Notably, the majority of cases emanated from socio-economic groups with comparatively lower levels of education, with 80% being male. Strikingly, 90% of the cases were found to be in the middle to late stages of infection based on CD4+ T cell counts. Among the 30 different serum antibody profiles examined, reactivity with seven bands (p24, p31, gp41, p51, p66, gp120, and gp160) emerged as the most commonly observed WB pattern. The absence of specific bands, specifically p55 (17.44%), p39 (32.31%), and p17 (25.64%) were most frequent, with the detection frequency of p17 bands significantly reduced among cases in the AIDS and middle stages. An analysis of drug resistance genotypes indicated that, despite viral mutations conferring resistance to certain reverse transcriptase inhibitors, the first-line treatment regimen remained effective for patients in Meizhou. Notably, mutations resistant to protease inhibitors were infrequent (2.7%), suggesting that incorporating protease inhibitors into the treatment regimen may enhance therapeutic outcomes for local patients. These findings provide essential insights into the specific epidemiological patterns, serum antibody profiles, and drug resistance genotypes of HIV-infected patients in Meizhou. Significantly, this research contributes to the formulation of future treatment strategies tailored to the local context.

## 1 Introduction

Human immunodeficiency virus (HIV) is the etiological agent responsible for acquired immune deficiency syndrome (AIDS). Since its discovery in 1983, HIV/AIDS has emerged as a global public health menace (Barre-Sinoussi et al., 1983). To date, an alarming 40.1 million individuals have succumbed to AIDS-related ailments since the onset of this epidemic (UNAIDS, 2022). As of 2021, approximately 38.4 million people across the globe were living with HIV, with approximately 1.5 million new infections documented (UNAIDS, 2022). In China, the incidence of HIV infection and the subsequent progression to AIDS has exhibited a consistent upward trajectory each year. Furthermore, the number of AIDS-related fatalities has eclipsed those attributable to any other infectious diseases (Wang et al., 2011; Ma et al., 2017; Qiao et al., 2019). HIV can be categorized into two distinct subtypes: HIV-1 and HIV-2 (Kapoor and Padival, 2022), with the former being responsible for > 95% of global infections (Duri et al., 2011). The progression of HIV infection can be stratified into three stages: acute HIV infection, clinical latency, and AIDS. The latter is characterized by a blood CD4 cell count of < 200 cells/mm^3^ (Kabir et al., 2020). Several diagnostic strategies for HIV have been developed, including antigen-antibody- and nucleic acid amplification-based assays (Kabir et al., 2020). Among these, western blotting (WB) stands as the gold standard for test validation due to its superior specificity (Gürtler, 1996; Dax and Arnott, 2004). The WB assay detects antibodies in the serum that interact with fixed HIV antigens. HIV structural proteins are encoded by the env, gag, and pol genes. The env gene encodes the envelope protein (gp160), which is subsequently cleaved into the mature surface protein (gp120) and the transmembrane protein (gp41) (Checkley et al., 2011). The Gag gene encodes polyprotein Gag (p55) and the cleaved Gag protein products consist of matrix protein (p17), capsid protein (p24), nucleocapsid protein (p7), and p6 (Sundquist and Kräusslich, 2012). A p39 fragment from Gag can also be detected by WB. Pol accounts for three enzymes: protease (PR, p10), reverse transcriptase (RT, p66/p51 heterodimer), and integrase (p31) (Sundquist and Kräusslich, 2012), which are the principal targets for antiviral drugs. WB profile analysis generates specific information on the HIV-specific serum antibody (Abs) in patients. Notable differences in the timing and intensity of antibody bands have been observed at different stages of HIV infection. While antibodies targeting the env antigen have been reported to appear in all clinical stages (Lange et al., 1986; Lange et al., 1987; Duri et al., 2011), antibodies to p17, p24, and its precursor p55 emerge post-infection and tend to diminish with the onset of clinical symptoms (Lange et al., 1987).

Presently, highly active antiretroviral therapy (HAART) represents the conventional clinical approach for managing AIDS. Since viral reverse transcriptase (RT) lacks proofreading capabilities, mutations occur in viral genes over time. HIV-1 reverse transcriptase (RT) is a major enzyme involved in HIV-1 replication. It has the synthesis function of DNA polymerase, but it does not have the correction function of DNA polymerase. Therefore, the viral nucleic acid shows a high error tendency during the replication of HIV-1. HIV-1 genetic variation is the result of errors in the replication of HIV-1 reverse transcriptase (RT) at a high rate. Recombinant virus occurs when more than one virus variant infects the same cell at the same time, and previral changes accumulate in the process of virus infection. Mutations in the RT and/or protease (PR) regions of progeny viruses may confer drug resistance, significantly reducing the efficacy of HAART and complicating current antiviral treatment strategies (Villar et al., 1995). Drug-resistant strains may vary among different regions and populations; hence, drug resistance genotype analysis is imperative for devising tailored treatment strategies.

Meizhou holds a significant status as a settlement for the Hakka population, an ethnic group in South China. Distinct AIDS prevalence characteristics in Meizhou compared to other regions are anticipated. In this study, we investigate and provide specific epidemiological patterns, serum antibody profiles, and drug resistance genotypes in HIV-infected individuals in Meizhou, China. This study systematically analyzed and reported the regional characteristics of HIV epidemiology and drug resistance in Meizhou city, based on the laboratory diagnostic and monitoring indicators of HIV infected population and the relevant situation of drug resistance. In order to explore the reasons for the failure of anti-HIV/AIDS in this region, it is very necessary to monitor the drug resistance of HIV-infected people, which is conducive to understanding the drug resistance of HIV-infected people in different regions, helping to update the treatment strategy, and providing a scientific basis for the prevention and control of AIDS in this region. However, the current drug resistance detection method still has some limitations, the absence of drug-resistant virus strains in plasma cannot be completely sure that there are no drug-resistant virus strains in HIV-infected people, which is a direction for our researchers to study in the future.

## 2 Materials and methods

### 2.1 Study population and screening

A total of 186257 serum samples from suspected patients, collected between May 2008 and December 2019, were obtained from Meizhou People’s Hospital. These samples underwent preliminary screening for HIV positivity, followed by confirmation through WB. This study was performed in accordance with the ethical standards of the Declaration of Helsinki and approved by the Human Ethics Committees of Meizhou People’s Hospital and communicated through letter no. 2020-C-129.

#### 2.1.1 Primary screening for HIV-positive samples

All samples were screened by using an HIV antibody/antigen enzyme-linked immunosorbent assay (ELISA) kit (Wantai Bio-Pharm) and HIV Ag/Ab Combo Reagent Kit (Abbott Germany Limited Partnership, USA). The protocol was performed according to the manufacturer’s instructions. In the ELISA test, 20μL of biotin-conjugate reagent and 100μL of the sample were added to a microwell strip pre-coated with recombinant HIV antigen and anti-p24 monoclonal antibody. The ELISA plate was incubated for 60 minutes at 37°C, followed by five washes. Next, 100 μL enzyme-labeled Ab/Ag was added for further incubation at 37°C for 30 minutes. After five washes, the substrate was added to the well for 30 minutes to allow for colour development, and the reaction was terminated by adding a stop solution. The optical density value of each well was measured using a microplate reader at a single wavelength of 450 nm or a dual wavelength of 450/630 nm. Chemiluminescence particle immunoassay was used to qualitatively detect the HIV p24 antigen and HIV-1/2 antibody (Abbott i2000SR, Abbott Laboratories, USA).

#### 2.1.2 Confirmation of HIV-positive samples with western blot test

The WB test was performed to detect antibodies in serum using an HIV Blot 2.2 kit (11039-036, MP Biomedical Asia Pacific Pte Ltd, Singapore), following the manufacturer’s instructions. Working solutions were prepared and placed at the corresponding pipette positions in a TECAN automated immunoblotting instrument. Nitrocellulose membrane strips carrying antigens to HIV-1/2 were soaked in washing buffer at a temperature between 22–28°C for 5 minutes, and then the liquid was removed. Two millilitres of immunoblotting buffer and 20μL of the sample were added to each strip, which was then placed in the TECAN automated immunoblotting instrument for detection. The WB results were interpreted according to the National AIDS Testing Technical Specification (Revised Edition 2015) of the China National Center for Disease Control and Prevention. The absence of a specific band or the presence of only the p17 band was considered a negative test result for the sample, while the presence of two env bands and one gag (or pol) band was considered a positive test result. Samples with insufficient bands on the membrane strip for a conclusive positive verdict were classified as uncertain specimens.

### 2.2 Flow cytometry analysis of CD4^+^ and CD8^+^ T cells

Blood cells were stained with the leukocyte differentiation antigen CD3/CD8/CD45/CD4 detection kit (340499, BD Bioscience, USA). Briefly, 10μL of fluorescein-labeled monoclonal antibodies were mixed and incubated with 25μL of EDTA-K2 anti-coagulated blood for 15 minutes. Erythrocyte lysis buffer (450μL) was added for another 15 minutes before the samples were analyzed by flow cytometry.

### 2.3 Analysis of drug-resistant genotypes in HIV-positive samples

HIV-infected blood samples were sent to the Guangdong AIDS Prevention Center for genotypic resistance testing. In brief, cDNA containing the protease-reverse transcriptase (PR-RT) sequence of HIV-1 was amplified and subjected to high-throughput sequencing. Sequences were submitted to the Stanford University HIV resistance database (http://hivdb.stanford.EDU) for subtype identification, resistance loci, and drug sensitivity analysis.

### 2.4 Statistical analysis

Data were analyzed using Statistical Package for the Social Sciences (SPSS) software, version 21.0. The student’s t-test was used to calculate *p* values, with statistical significance set at *p* < 0.05.

## 3 Results

### 3.1 HIV screening of serum samples in the Meizhou region

**Figure 1:**
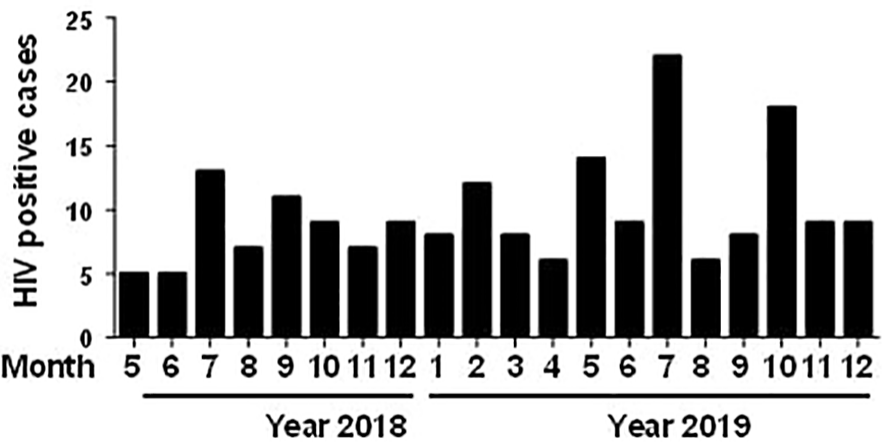
Distribution of 195 confirmed HIV-positive cases by month.

Of the 186,257 samples screened, 210 (0.11%) were positive using ELISA, and 291 (1.94%) were positive using chemiluminescence immunoassay (CLIA). To eliminate false-positive results in preliminary screening, 281 preliminary positive samples underwent further validation through western blotting, with 195 samples confirmed as positive (positive ratio = 69.40%). Among the 195 HIV-infected individuals, 157 (80.51%) were male, and 38 (19.49%) were female (**Table 1**). Regarding age distribution, 1 (0.51%) was diagnosed between 0 and 18 years, 32 (16.41%) between 19 and 30 years, 55 (28.21%) between 31 and 45 years, 54 (27.69%) between 46-60 years, 48 (24.62%) between 61 and 80 years, and 5 (2.56%) were over 80 years of age (**Table 1**). The youngest patient was 18 years old, and the oldest was 85. Of the HIV-infected cases, 151 cases (77.44%) were permanent residents of Meizhou, and 44 cases (19.49%) came from other areas (**Table 1**). Students and government personnel accounted for 5.13% (10 cases), farmers and workers for 76.92% (150 cases), and others for 17.95% (35 cases) (**Table 1**). The distribution of the 195 confirmed samples is presented monthly based on the sampling period (**Figure 1**). A comparison of cases from May to December (5–12) in 2018 and 2019 showed a higher number of HIV-infected individuals in 2019. An analysis of monthly cases revealed the highest incidence of HIV diagnoses in July of each year. The CD4^+^ T cell numbers were further determined for 155 HIV- positive samples, following which the cases were classified into different clinical stages (Liu et al., 2020): the primary infection stage (15 cases, 9.7%), the middle stage of infection (63 cases, 40.6%), and AIDS stage (77 cases, 49.7%) (**Supplementary Table S1**). There was a significant difference in the CD4^+^ T lymphocyte values at different clinical stages (*p* < 0.01). The statistical results show that the majority of HIV-infected patients in Meizhou were in the middle of the AIDS stages of infection.

**Table 1:** Basic characteristics of 195 HIV-infected patients [No. of cases (%)].

Age	0-18	19-30	31-45	46-60	61-80	>80	Total	χ^2^	*p* value
**Sex**								3.21	>0.05
Male	1 (0.51)	27 (13.85)	43 (22.05)	42 (21.54)	39 (20)	5 (2.56)	157 (80.51)		
Female	0 (0)	5 (2.56)	12 (6.15)	12 (6.15)	9 (4.61)	0 (0)	38 (19.49)		
**Population area**								20.42	<0.01*
Native	1 (0.51)	23 (11.79)	33 (16.92)	47 (24.10)	43 (22.05)	4 (2.05)	151 (77.44)		
Migrant	0 (0)	9 (4.61)	22 (11.28)	7 (3.59)	5 (2.56)	1 (0.51)	44 (22.56)		
**Occupation**								27.2	<0.01*
Students& government personnel	1 (0.51)	4 (2.05)	1 (0.51)	1 (0.51)	2 (1.03)	1 (0.51)	10 (5.13)		
Farmer/worker	0 (0)	20 (10.26)	46 (23.59)	41 (21.03)	41 (21.03)	2 (1.03)	150 (76.92)		
Others	0 (0)	7 (3.59)	8 (4.10)	12 (6.15)	6 (3.08)	2 (1.03)	35 (17.95)		

*, indicates a statistically significant difference.

### 3.2 Characteristics of Western blot profiles in HIV infection

**Figure 2:**
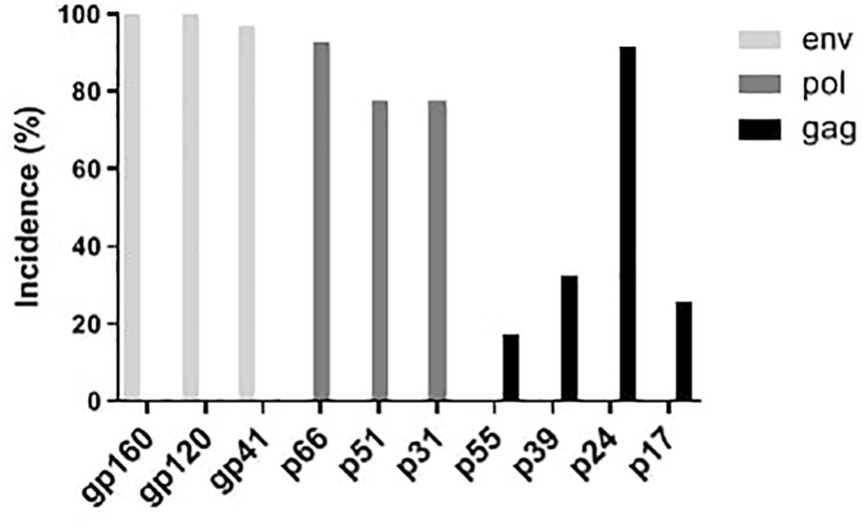
Distribution of western blot bands of serum antibodies against HIV proteins from 195 HIV positive samples.

WB results of the 195 HIV-1 positive samples were analyzed and classified into 30 types of patterns (**Table 2**). Reactivity with seven bands (p24, p31, gp41, p51, p66, gp120, and gp160) was the most commonly observed WB pattern, with a proportion of 28.21% (55/195), while the complete 10-band pattern occurred in only 9.74% (19/195) of the samples. Of the 195 samples, 133 were full band or sub-full band (over 7 bands) patterns, accounting for 67.99%. The percentage distribution of the specific bands in the investigated samples is presented in **Figure 2**. The three envelope protein bands (gp160, gp120, and gp41) were the most prevalent, with ratios of 100%, 100%, and 96.92%, respectively, followed by p66 (92.82%) and p24 (91.79%). In contrast, p55 (17.44%), p39 (32.31%), and p17 (25.64%) were the most frequently missing bands (**Figure 2**). The detection rates of p55 and p39 were low in each clinical stage, while the p17 bands that were significantly lower in cases at the middle stages and AIDS stages of the infection (**Table 3**).

**Table 2:** Western blot patterns of 195 HIV positive samples.

Type	Sample amount	Percentage (%)	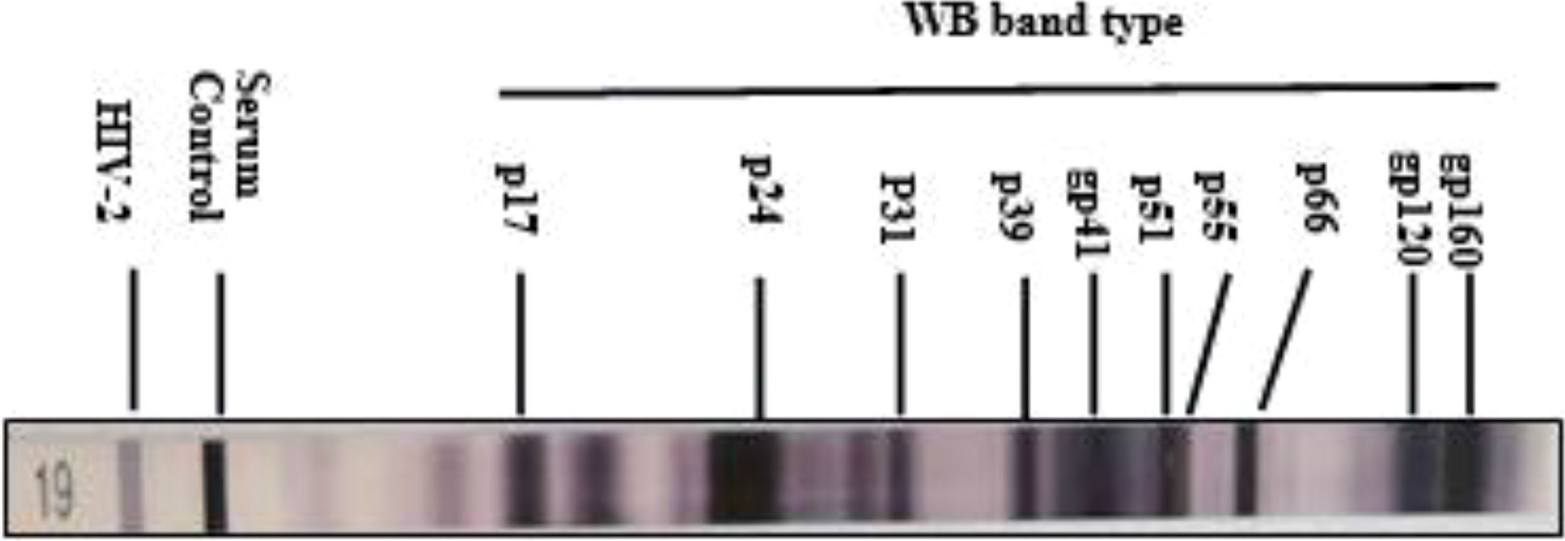													
1	55	28.21				–		+	+	–	+	+	–	+	+	+
2	19	9.74				+		+	+	+	+	+	+	+	+	+
3	14	7.18				+		+	+	+	+	+	–	+	+	+
4	13	6.67				–		+	–	–	+	+	–	+	+	+
5	12	6.15				–		+	+	+	+	+	–	+	+	+
6	9	4.62				–		+	+	+	+	+	+	+	+	+
7	3	1.54				+		+	+	–	+	+	+	+	+	+
8	8	4.08				+		+	+	–	+	+	–	+	+	+
9	3	1.54				–		+	+	–	+	+	+	+	+	+
10	1	0.51				+		+	+	+	+	–	–	+	+	+
11	4	2.05				–		+	–	+	+	+	–	+	+	+
12	2	1.03				+		+	–	+	+	–	–	+	+	+
13	2	1.03				+		+	–	–	+	+	–	+	+	+
14	1	0.51				–		+	+	+	+	–	–	+	+	+
15	9	4.62				–		+	+	–	+	–	–	+	+	+
16	6	3.08				–		–	–	+	+	+	–	+	+	+
17	1	0.51				–		+	–	+	+	–	–	+	+	+
18	9	4.62				–		+	–	–	+	–	–	+	+	+
19	6	3.08				–		+	+	–	+	–	–	–	+	+
20	4	2.05				–		–	+	–	+	–	–	+	+	+
21	2	1.03				–		–	–	–	+	+	–	+	+	+
22	1	0.51				–		+	–	–	–	+	–	+	+	+
23	1	0.51				+		+	–	–	+	–	–	–	+	+
24	2	1.03				–		+	–	–	+	–	–	–	+	+
25	1	0.51				–		–	–	–	–	+	–	+	+	+
26	1	0.51				–		–	–	–	+	–	–	+	+	+
27	1	0.51				–		–	+	–	+	–	–	–	+	+
28	1	0.51				–		+	–	–	–	–	–	+	+	+
29	3	1.54				–		+	–	–	–	–	–	–	+	+
30	1	0.51				–		–	–	–	+	–	–	–	+	+

**Table 3:** Association of clinical stage to different western blot bands in 155 patients of Meizhou who tested positive for HIV-1 antibodies between 2018 and 2019.

WB bands	Primary infection		Middle stage		AIDS stage		X^2^	
	Cases	Ratio (%)	Cases	Ratio (%)	Cases	Ratio (%)		*p* value
gp160	15	100	63	100	77	100	/	/
gp120	15	100	63	100	77	100	/	/
P66	15	100	60	95.2	69	89.6	2.93	>0.05
P55	4	26.7	12	19	12	15.6	1.11	>0.05
P51	12	80	50	79.4	58	75.3	0.39	>0.05
gp41	14	93.3	61	96.8	74	96.1	0.40	>0.05
P39	8	53.3	24	38.1	19	24.7	5.97	>0.05
P31	12	80	50	79.4	58	75.3	0.39	>0.05
P24	15	100	60	95.2	66	85.7	5.47	>0.05
P17	9	60	11	17.5	22	28.6	11.27	<0.01*

*, indicates a statistically significant difference.

### 3.3 Drug resistance and genomics

HIV-1 genetic mutation and drug resistance are major barriers to successful antiretroviral therapy. To elucidate the HIV-1 genotype drug resistance characteristics in HIV-infected people of Meizhou, serum samples from 37 untreated patients [29 men (78%); 8 women (22%)] obtained from Meizhou People’s Hospital were included. The average age of the patients was 44 years (ranging from 7 to 80 years). The patients were infected through various routes: heterosexual transmission (75.68%, 28/37), homosexual transmission (8.11%, 3/37), mother-to-child transmission (2.70%, 1/37), and unclear transmission (13.51%, 5/37). The most prevalent HIV-1 subtype was circulating recombinant form CRF01_AE (64.86%, 24/37), followed by CRF07-BC (10.81%, 4/37), B+CRF01-AE (5.40%, 2/37), CRF55-01B (5.40%, 2/37), B+C (5.4%, 2/37), A (2.70%, 1/37), B (2.70%, 1/37), and C (2.70%, 1/37). The initial antiviral treatment regimen consisted of two nucleoside reverse transcriptase inhibitors (NRTIs) plus efavirenz (EFV)/nevirapine (NVP) in 31 cases (83.79%) or zidovudine (AZT)/lamivudine (3TC) plus EFV in 6 cases (16.21%). Pro-RT gene sequences from the samples were obtained and analyzed for drug resistance-related mutations. Among the 37 PCR-positive cases, 23 carried one or more drug-resistance mutations. The drug resistance mutations included 10 mutations for NRTIs, with M184V/I (15 cases) as the main mutation site, 11 mutations for non-nucleoside reverse transcriptase inhibitors (NNRTIs), with K103KN (12 cases) as the main mutation site, and four resistant mutations for protease inhibitors (PIs) (**Table 4**). The RT inhibitor resistance rate was 62.16% (23/37) compared to 2.7% (1/37) for PIs, indicating that RT inhibitor resistance is the prevalent mutation in Meizhou. Moreover, cross-resistance between NRTIs and NNRTIs co-occurred in patients, accounting for 40.54% of drug-resistant mutations. Based on the mutations carried by each HIV-infected individual, their sensitivity to anti-HIV drugs used in clinical practice was further assessed (**Supplementary Table S2**).

**Table 4:** HIV-1 resistant mutations in samples from 37 HIV-1 infected patients from Meizhou.

NRTIs		NNRTIs		PIs	
Mutation site	Cases	Mutation site	Cases	Mutation site	Cases
M184V/I	15	K103KN	12	M46I	1
K70A/E/K/R/KE	6	V106I/M/VI	6	I54IV	1
K65R	4	V179D/E/EQ	5	L76V	1
D67N/G/DN	5	G190A/Q/S	4	V82A	1
M41ML	2	K101E/H	2		
L74LI	3	E138G/EQ	2		
T215TFIS	1	Y181C/V	4		
K219Q	1	P225H	3		
Y115F	2	V108I/VI	3		
V75VIM	1	M230ML	1		

NRTIs, nucleoside reverse transcriptase inhibitors; NNRTIs, non-nucleoside reverse transcriptase inhibitors; PIs, protease inhibitors. The four resistant mutants to PIs were simultaneously found in one case.

## 4 Discussion

In this study, serum samples collected from the HIV Confirmatory Laboratory of Meizhou People’s Hospital Medical Laboratory Center (From May 2008 to December 2019) were analyzed. Primary HIV screening by ELISA or CLIA revealed HIV-positive rates of 0.11% and 1.94%, respectively. ELISA and CLIA are simple and high-throughput; however, they are associated with relatively high false-positive outcomes (Liu and Jiang, 2004; Chen, 2012; Wu et al., 2018). More rigorous strategies, such as western blotting, are needed for verification. Herein, 195 samples were confirmed as HIV-1 positive by western blotting, with no HIV-2 positive samples being observed. Most HIV-1 infected patients were determined to be in the middle and AIDS stages of the infection (**Supplementary Table S1**), and there were more men than women patients (**Table 1**). The HIV-1 infected patients are mainly farmers and migrant workers (**Table 1**), whose education level is generally low, and who may have less knowledge about AIDS prevention. It is particularly important to further enhance the coverage of AIDS prevention knowledge in the rural population. The highest detection rate of HIV-infected was in July (**Figure 1**); nonetheless, the reason behind this observation remains to be further investigated. A contemporaneous comparison of HIV-diagnosed cases in 2018 and 2019 suggested an increasing trend in Meizhou (**Figure 1**). Therefore, HIV prevention and control in the Meizhou community cannot be relaxed. It is necessary to further improve the coverage of HIV screening to achieve early detection and treatment. WB is the gold standard for confirming HIV infection and provides information on virus and host responses. The full WB pattern contains 10 bands that are specific to HIV-1. However, the band patterns of WB vary according to stages of the infection, prevalent viral strains, and cohorts, thus displaying significant regional characteristics. In this study, we observed 30 types of WB patterns from the 195 HIV-positive samples (**Table 2**). Reactivity with seven bands (p24, p31, gp41, p51, p66, gp120, and gp160) was the most commonly observed pattern with a proportion of 28.21%, while the complete 10-band pattern occurred in only 9.74% of the samples, which was different from other studies that reported the full pattern as the main outcome (Sudha et al., 2006). Envelope protein bands (gp160, gp120, and gp41), pol protein (p66), and gag protein (p24) were the most prevalent, which was consistent with the previous studies (Zhong et al., 2012; Xie and Chen, 2018). p55, p39, and p17 were the most frequently missing bands (**Figure 2**). The frequencies of p55 and p39 bands were low in samples from every infection stage (**Table 3**), but the p17 bands were only significantly lower in cases at the AIDS and middle stages of the infection (**Table 3**). These results indicated that lack of antibody reactivity to p17 was associated with disease progression. HAART is the most effective clinical treatment for AIDS (Sattwika et al., 2023). In Meizhou, TDF/AZT +3TC+EFV/NVP is the most commonly adopted initial treatment regimen, with the EFV+AZT/3TC regimen being less prescribed. Based on the drug-resistant genotype assay results, the main resistant HIV-1 strains in Meizhou were the CRF01-AE subtype, followed by the CRF07-BC subtype, which is consistent with the current situation in China (Huo et al., 2019).

In Meizhou, the resistance mutation of NNRTIs is the most prevalent, followed by that of NRTIs. Moreover, the cross-resistance of NRTIs and NNRTIs co-occurred in patients and accounted for 40.54% of drug-resistant mutations, indicating that multidrug resistance is widespread in the region. This may be related to the long-term use of first-line therapeutic drugs, resulting in the continuous accumulation of cross-drug-resistant mutant HIV-1 strains (Ma et al., 2010). Studies have shown that the accumulation of thymidine analogue-resistant mutations causes almost all NRTI resistance (Zha and Zha, 2006). In the resistance mutations of NRTIs, the M184V mutation was most prevalent, followed by K65R (**Table 4**). It has been reported that the M184V mutation causes significant resistance to 3TC but increases the sensitivity to TDF and AZT (Zuo, 2020). The K65R mutation was reported to produce moderate or high resistance to TDF and ABC (Guo, 2014). In this study, we observed that some patients simultaneously harboured mutations at the K65R and M184V sites; however, treatment with AZT was still appropriate, indicating that the first-line treatment regimen was efficacious for patients in Meizhou with this drug-resistant form of the disease. These results may explain why the HIV antiviral treatment failure ratio due to resistant mutations in Meizhou (8.95%) was much lower than the global data (40.30%–84.00%) (Shen and Su, 2019). Compared with the high incidence of drug-resistant mutations in NRTIs and NNRTIs, resistant mutations in PIs were only found in one out of the 37 cases (**Table 4**). These results suggest that patients in Meizhou with drug-resistant HIV/AIDS may still be sensitive to PIs since resistance mutations rarely occur in response to this group of drugs. Therefore, when patients develop cross-resistance to NRTIs and NNRTIs simultaneously and the first-line treatment regimen ceases to be efficacious, second-line treatment may be adopted. The study of clinical and epidemiological characteristics of HIV patients is conducive to the establishment of regional HIV transmission and epidemic identification standards and early warning systems. At the same time, the correlation between serological characteristics and clinical drug resistance was analyzed to provide useful information for clinical timely acquisition of HIV biological information and prediction of drug resistance (Shchemelev et al., 2023). The analysis model of the correlation between serotype and drug resistance may provide new ideas for HIV epidemic prevention and identification analysis. By studying the laboratory diagnosis and monitoring indicators and drug resistance of HIV infected people in Meizhou city, we provide scientific clinical basis for AIDS prevention and treatment. Although the detection methods of HIV infection are becoming more and more mature and diversified, there are still some problems that need to be studied and solved. One of the prominent problems is the long incubation period of HIV-infected people and the different window periods of HIV detection methods. We combine the characteristics of the regional HIV epidemic in the detection population and adopt appropriate detection methods to reduce the missed detection rate of HIV-infected people. In the process of antiretroviral drug treatment, clinical laboratory detection of drug resistance genes appears in various problems, we need to further explore, such as how long the interval of drug resistance monitoring detection is appropriate, can timely understand the characteristics of HIV antiviral resistance, is conducive to HIV infected people to develop effective treatment plans.

## 5 Conclusion

In summary, we explored the characteristics of clinical HIV infection in Meizhou, China, by retrospectively analyzing samples from HIV-infected people obtained from May 2008 to December 2019. Most of the 195 positive cases were found to be male and, in the middle, and AIDS stages. They were mainly from groups with relatively lower education levels. We found that the most prevalent pattern was reactivity with seven bands (p24, p31, gp41, p51, p66, gp120, and gp160), instead of the full-band pattern reported in other regions. The WB bands including p55, p39, and p17 were the most frequently missing bands. We further observed decreased detection of the p17 antibody in patients in the middle to the AIDS stage of the infection/disease. Most resistant mutations were related to NRTIs and NNRTIs; only one case carried a mutation resistant to PIs, suggesting the presence of protease inhibitors in the treatment regimen will work better in local patients. Our data described the specific epidemiological patterns, serum antibody profiles and drug resistance genotypes of HIV-infected patients in Meizhou, and are of great significance for guiding local treatment strategies in the future.

## Data availability statement

The original contributions presented in the study are included in the article/**Supplementary Material**. Further inquiries can be directed to the corresponding authors.

## Ethics statement

This study was performed in accordance with the ethical standards of the Declaration of Helsinki and approved by the Human Ethics Committees of Meizhou People’s Hospital and communicated through letter no. 2020-C-129.

## Author contributions

XL: Conceptualization, Data curation, Project administration, Visualization, Writing – original draft. SP: Investigation, Project administration, Resources, Supervision, Writing – review & editing. XG: Funding acquisition, Investigation, Methodology, Project administration, Writing – original draft. FW: Data curation, Funding acquisition, Software, Writing – review & editing. XZ: Conceptualization, Formal Analysis, Funding acquisition, Investigation, Writing – original draft. ZZ: Conceptualization, Formal Analysis, Funding acquisition, Visualization, Writing – original draft. XW: Conceptualization, Methodology, Resources, Supervision, Writing – original draft.
