# Supplementary material for: Clinical, epidemiological, and drug resistance insights into HIV-positive patients in Meizhou, China
Source: Front Cell Infect Microbiol. 2024 Jan 19;13:1330826. doi: 10.3389/fcimb.2023.1330826 (PMC10835272; doi:10.3389/fcimb.2023.1330826)
Supplement: Supplementary file 1 [file Table_1.docx]

***Supplementary Material***

**Table S1 Clinic stages of the 155 HIV-1 antibody positive patients in Meizhou**

| Group | Cases | Ratio (%) | CD4^+^ T cell number/μl |
| --- | --- | --- | --- |
| Primary infection stage | 15 | 9.7 | 668.53 ± 201.70 |
| Middle stage of infection | 63 | 40.6 | 328.48 ± 85.30 |
| AIDS stage | 77 | 49.7 | 65.40 ± 63.41 |

**Table S2 Case number of antiviral drug resistance in HIV/AIDS patients from Meizhou region**

| Drugs | Sensitive |  | Resistant | | | | | | |
| --- | --- | --- | --- | --- | --- | --- | --- | --- | --- |
|  |  |  | Potential |  | Low |  | Moderate |  | High |
| NRTIs |  |  |  |  |  |  |  |  |  |
| Abacavir (ABC) | 14 |  | 0 |  | 5 |  | 3 |  | 8 |
| Zidovudine (AZT) | 28 |  | 0 |  | 0 |  | 0 |  | 2 |
| Stavudine (D4T) | 20 |  | 0 |  | 1 |  | 7 |  | 2 |
| Didanosine (DDI) | 14 |  | 3 |  | 3 |  | 1 |  | 9 |
| Emtriva (FTC) | 14 |  | 1 |  | 0 |  | 0 |  | 15 |
| Lamivudine (3TC) | 14 |  | 1 |  | 0 |  | 0 |  | 15 |
| Tenofovir (TDF) | 20 |  | 0 |  | 5 |  | 3 |  | 2 |
| NNRTIs |  |  |  |  |  |  |  |  |  |
| Doravirine (DOR) | 15 |  | 1 |  | 4 |  | 5 |  | 2 |
| Efavirenz (EFV) | 9 |  | 1 |  | 2 |  | 0 |  | 18 |
| Etravirine (ETR) | 14 |  | 7 |  | 2 |  | 5 |  | 2 |
| Nevirapine (NVP) | 8 |  | 2 |  | 0 |  | 0 |  | 20 |
| Rilpivirine (RPV) | 14 |  | 4 |  | 5 |  | 2 |  | 5 |
| PIs |  |  |  |  |  |  |  |  |  |
| Atazanavir (ATV) | 29 |  | 0 |  | 0 |  | 0 |  | 1 |
| Darunavir (DRV) | 29 |  | 0 |  | 1 |  | 0 |  | 0 |
| Fosamprenavir (FPV) | 29 |  | 0 |  | 0 |  | 0 |  | 1 |
| Indinavir (IDV) | 29 |  | 0 |  | 0 |  | 0 |  | 1 |
| Lopinavir (LPV) | 29 |  | 0 |  | 0 |  | 0 |  | 1 |
| Nelfinavir (NFV) | 29 |  | 0 |  | 0 |  | 0 |  | 1 |
| Saquinavir (SQV) | 29 |  | 0 |  | 0 |  | 0 |  | 1 |
| Tipranavir (TPV) | 29 |  | 0 |  | 0 |  | 1 |  | 0 |

NRTIs: nucleoside reverse transcriptase inhibitors; NNRTIs: non-nucleoside reverse transcriptase inhibitors; PIs: protease inhibitors.
